# Etiology and Prognostic Criteria for Liver Failure in Southeast China: A Multicenter Retrospective Cohort Study Between 2018 and 2020

**DOI:** 10.1155/grp/5512889

**Published:** 2024-12-18

**Authors:** Chunyan Lyu, Jun Han, Naling Kang, Dawu Zeng, Chantsalmaa Davgadorj, Lina Ge, Meifang Zhou, Richeng Mao, Yan Yan

**Affiliations:** ^1^Clinical Medical Research Center, The Fifth People's Hospital of Wuxi, Wuxi, China; ^2^Clinical Medical Resarch Center, Wuxi Clinical College of Nantong University, Wuxi, China; ^3^Department of Infectious Diseases, The Fifth People's Hospital of Wuxi, Wuxi, China; ^4^Department of Infectious Diseases, The First Affiliated Hospital of Fujian Medical University, Fuzhou, Fujian, China; ^5^Department of Infectious Diseases, Huashan Hospital Affiliated With Fudan University, Shanghai, China

**Keywords:** acute-on-chronic liver failure, antithrombin III, hepatitis B virus (HBV), liver failure, prognostic

## Abstract

**Background:** The prognosis of patients with liver failure (LF) depends significantly on the etiology and clinical indicators. This analysis of these basic indicators can help provide a basis for the study of predictive outcome indicators.

**Methods:** We collected the data from multiple centers in Southeast China, including subclasses of acute liver failure (ALF), subacute liver failure (SLF), acute-on-chronic liver failure (ACLF), subacute-on-chronic liver failure (SALF), and chronic liver failure (CLF). Multivariate logistic regression analysis was used to screen for clinical indicators of nonsurvivors. We analyzed receiver operating characteristic (ROC) curves and cutoff values to assess the prognostic criteria.

**Results:** Hepatitis B virus (HBV) infection is the leading etiology of patients with LF (64.52% (411/637)). SALF (41.36%) and CLF (32.30%) are the main subclasses of the hepatitis B virus–related liver failure (HBV-LF) group and the non-HBV–related LF group in Southeast China, respectively. Between 2018 and 2020, the incidence of HBV-LF decreased significantly, ranging from 72.36% to 59.74%, and the spontaneous survival rates of patients with HBV-LF were substantially lower than those of non-HBV-LF patients (36.43%~44.93% vs. 58.97%~63.64%). Infection and cirrhosis were the leading causes of death in both groups. The age and total bilirubin value of the nonsurvivors with HBV-LF were significantly higher, and the number of days of hospitalization was significantly shorter than that of the survivors. The ages of the nonsurvivors in the non-HBV-LF group were significantly higher than those of the survivors. The prothrombin time–international normalized ratio (PT-INR) is 2.05, 1.92, or 2.11, and antithrombin III (AT III) is 24.50%, which are proposed as prognostic criteria for the HBV-SALF (hepatitis B virus–related subacute-on-chronic liver failure), non-HBV-SLF (non-hepatitis B virus–related subacute liver failure), non-HBV-ACLF (non-hepatitis B virus–related acute-on-chronic liver failure), and HBV-ALF (hepatitis B virus–related acute liver failure) subclasses, respectively.

**Conclusions:** The incidence of HBV-LF is decreasing annually. AT III, as an independent prognostic criterion, has excellent discriminative ability for the outcomes of the HBV-ALF subclass.

## 1. Background

Liver failure (LF) is a clinical symptom of severe or chronic hepatic injury caused by various factors [1]. However, there has yet to be consensus on the definition, classification, and prognostic criteria of LF subclasses in different countries [2, 3]. For example, the classification system for acute liver failure (ALF) includes the O'Grady system, the Bernuau system, and the Japanese system; all initially define LF as a severe liver injury, which may be reversible, and in the absence of preexisting liver disease, hepatic encephalopathy may occur within 8 weeks after the initial symptoms [2]. Furthermore, a previous study reported that the prevalence and 30-day mortality rates in patients with acute-on-chronic liver failure (ACLF) determined by different criteria might be inconsistent worldwide [4].

Given that LF is a complex pathophysiological process caused by multiple etiologies, it is difficult for treatment guidelines to include or address all LF problems. Validation and comparative studies have shown that the European Consortium for the Study of Chronic Liver Failure (EASL-CLIF) criteria have better sensitivity for diagnostic and prognostic capacity, whereas the North American Consortium for the Study of End-Stage Liver Disease (NACSELD) criteria are highly accurate in predicting mortality [4, 5]. To date, the most reported severe hepatitis of ACLF was first proposed by Japanese scholars in 1995, and through expert consultations between the EASL-CLIF and the Asia–Pacific Association for the Study of the Liver (APASL), a consensus for ACLF was finally confirmed in 2018 [6]. On the basis of our observations, the research team proposed a diagnostic criterion for Chinese ACLF patients [7], almost identical to the APASL criteria [8].

The incidence of LF varies with geography and time [9]. The etiology shows large geographical differences among countries, such as in the United Kingdom and the United States, mainly due to acetaminophen-induced ALF. Moreover, hepatitis B virus (HBV) and hepatitis E virus (HEV) infections are the leading causes in Japan and India, respectively [2]. The incidence of HBV varies widely in the Asia–Pacific region, and China has the highest rate of HBV infection [10, 11]. The Chinese criteria divide LF into the ALF, subacute liver failure (SLF), ACLF, subacute-on-chronic liver failure (SALF), and chronic liver failure (CLF) subclasses, which are also feasible for the exploration of effective treatment strategies [6]. Xie et al. analyzed the etiological classification and outcome of ACLF in Southwest China from 2000 to 2012 according to the Chinese criteria [1]. The classification of LF differs from that of clinical laboratory data and liver biopsies [11, 12]. For example, there is only a single case of hepatic necrosis in acute severe hepatitis; however, there are repeated and continuous cases of hepatic necrosis in subacute severe hepatitis [11]. HBV is one of the leading causes of severe hepatitis in China, and efforts have been made to prevent severe hepatitis and adverse outcomes through medical treatment and vaccination control. Over the past two decades, with the use of birth-dose HBV vaccination and antiviral drugs in urban and rural communities in China, the incidences of HBV-related cirrhosis, cancer, and LF may have changed [10, 13, 14]. The incidence of LF caused by HBV in Southwest China is 91.6%, but the survival rate has increased annually [1]. You et al. reported that the incidence of LF caused by HBV in northern China ranged from 86.5% to 69.2% between 2002 and 2011 [14].

In recent years, there have been numerous reports on the etiologies of ALF and ACLF worldwide [9, 15–18]. However, there need more data concerning the incidence, etiology, or prognosis of various types of LF in Southeast China. Since 2018, the East Coastal Infectious Diseases Alliance of China has conducted annual surveillance of patients with LF according to the new diagnostic criteria [6]. In recent decades, bilirubin has been a prognostic biomarker for short-term survival in patients with ACLF, particularly for emerging interventions such as extracorporeal liver assist devices and early-phase improvements in pharmacological therapies [19]. On the one hand, clinical and biochemical markers of liver dysfunction, such as the Child–Pugh classification, the Model for End-Stage Liver Disease (MELD), and King's Standard (for ALF), analyze biochemical markers of liver synthesis function, such as total bilirubin (TBil), biliary globin and albumin, and the prothrombin time–international normalized ratio (PT-INR), to assess disease severity [19–22]. This study is aimed at elucidating the clinical pathogenesis of patients with hepatitis B virus–related liver failure (HBV-LF) and non-HBV-LF in a retrospective cohort study and developing new diagnostic or prognostic criteria for the subclassical classification of LF diagnoses, such as age; the number of days of hospitalization; and PT-INR, alanine aminotransferase (ALT), TBil, and antithrombin III (AT III) levels. The cutoff values of these indicators help determine the prognosis of patients with the LF subclass, help to promote etiological treatment, and provide a theoretical basis for diagnostic criteria.

## 2. Methods

### 2.1. Patient Collection

Retrospective cohort studies of LF were conducted through an annual survey of three liver centers in the southeastern coastal region of China, including three general tertiary hospitals in Wuxi, Shanghai, and Fujian. The medical records and demographic information of hospitalized patients with LF from January 2018 to December 2020 were collected through computerized medical record system. The inclusion criteria were as follows: (1) patients whose LF diagnosis met the criteria of the “Guidelines for the Diagnosis and Treatment of Liver Failure (2018 Edition)” [6], (2) patients aged > 1 year, (3) patients who were hospitalized, and (4) survivors: The survival status of patients was investigated at the following time points: in the period of hospitalization and 2 weeks and 6 months postdischarge. The survival rate was investigated via telephone follow-up postdischarge. The exclusion criteria were patients whose medical records lacked clinical or laboratory diagnostic information and who were lost to follow-up. This study was conducted in accordance with the Declaration of Helsinki (as revised in 2013) and approved by the Fifth People's Hospital of Wuxi Clinical Research Ethics Committee (No. 2021-007-1).

### 2.2. Etiologies and Trigger Factors

Demographic data, such as the HBV-LF and non-HBV-LF patients' etiological indicators, include sex, age, comorbidities, PT-INR, TBil, AT III values, the number of days of hospitalization, the incidence of hepatic encephalopathy, the number of patients who died during treatment or 2 weeks postdischarge, and the number of patients who survived 6 months postdischarge. In addition, data of spontaneous survival (SS) rate (the survivors/total patients per year) was obtained and calculated from patients or legal surrogates through telephone follow-up on 2 weeks or 6 months [1]. The incidence of LF represents the frequency of disease caused by a particular cause in a population during a given year. The trigger factors of the HBV-LF and non-HBV-LF included the following events: hepatitis A–E, Wilson's disease, liver cancer, cirrhosis, pharmaceuticals, alcohol, autoimmune hepatitis, diabetes, primary biliary cirrhosis (PBC), and Schistosome infection.

### 2.3. Statistical Analysis

GraphPad Prism 8.0.1 statistical software was used for data analysis. The population variables' frequency, distributions, and clinical outcomes were described proportionally, and the differences in the biochemical markers between the two groups were analyzed using the mean ± standard deviation (*X* ± SD). Differences in the biochemical markers between the two groups were analyzed via Student's *t*-test, the Wilcoxon signed-rank test, and the Mann–Whitney test. Count data were analyzed via chi-square tests or rates of occurrence (in percentages). The risk factors affecting death from LF were analyzed via multiple logistic regression via SPSS Statistics 22.0 software. The prognostic value of each clinical indicator for LF was analyzed via receiver operating characteristic (ROC) curve and area under the curve (AUC) analyses. In all calculations, *p* values less than 0.05 were considered statistically significant.

## 3. Results

### 3.1. Proportions of Patients With LF in Southeast China

**Figure 1:**
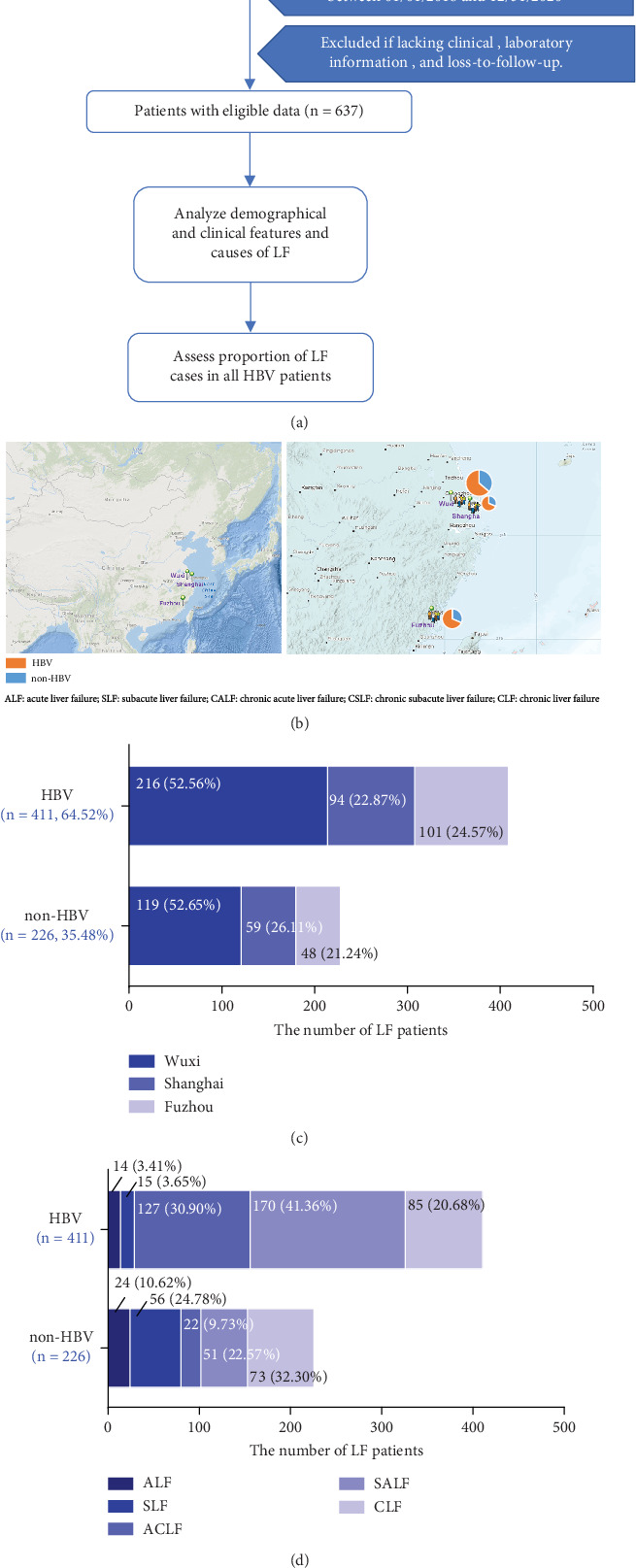
Proportion of liver failure (LF) subclasses in southeastern China. (a) Screening process for patients with LF. (b) Composition of the hepatitis B virus (HBV) group and the non-HBV group from three centers between 2018 and 2020. (c) Geographical distribution of patients with liver failure in the three centers. (d) Proportions of the HBV-LF and non-HBV-LF subclasses. ALF, acute liver failure; SLF, subacute liver failure; ACLF, acute-on-chronic liver failure; SALF, subacute-on-chronic liver failure; CLF, chronic liver failure.

Between 2018 and 2020, we collected the clinical and demographic information of 689 patients with LF at three designated centers in Southeast China. We removed patients with LF who met the exclusion criteria, including 2 patients with LF whose hospitalization days were outside of the study time frame, 3 patients with LF due to incomplete clinical data, 47 patients with LF due to loss to follow-up at 2 weeks and 6 months after discharge from the hospital (*n* = 52), and the remaining 637 patients with LF were eligible (Figure 1(a)). After clinical and demographic analysis, HBV-LF was the dominant cause in patients with LF at each center (Figures 1(b) and 1(c)). Among the 637 patients, the number of patients in the HBV-LF group was significantly higher than that in the non-HBV-LF group (~1.82-fold, 64.52% (411/637) vs. 35.48% (226/637)). According to the subclass classification, the three main subclasses of the HBV-LF group were SALF (41.36%), ACLF (30.90%), and CLF (20.68%). The three main subclasses in the non-HBV-LF group were CLF (32.30%), SLF (24.78%), and SALF (22.57%) (Figure 1(d)). Therefore, HBV infection was the dominant cause of LF in Southeast China. The dominant subclasses of the two groups were the hepatitis B virus–related subacute-on-chronic liver failure (HBV-SALF) and non-hepatitis B virus–related chronic liver failure (non-HBV-CLF) subclasses.

### 3.2. Incidences and SS Rates of LF

The incidence and SS rates and the proportions of ALF, SALF, and ACLF diagnoses changed annually between 2000 and 2012 in Southwest China [1]. In our retrospective cohort study, the incidence of HBV-LF ranged from 72.36% to 59.74% between 2018 and 2020. Compared with that in 2018, the incidence of HBV-LF decreased significantly between 2019 (*p* < 0.001) and 2020 (*p* < 0.01). After the survival information of discharged patients (2 weeks) was obtained, the SS rate in the HBV-LF group reached 44.93% in 2019, and it reached 63.64% in the non-HBV-LF group in 2018. Compared with the non-HBV-LF group, the HBV-LF group presented significantly lower SS rates between 2018 and 2020, and the number of patients with ACLF and SALF in the HBV-LF group was 2.50~8.50-fold higher. The number of patients with CLF that occurred in both groups was similar. In general, the results suggest that the incidence of HBV-LF is decreasing annually and that the incidence of ACLF and SALF is dominant (Table 1).

### 3.3. Etiological Component of the HBV-LF and Non-HBV-LF

**Figure 2:**
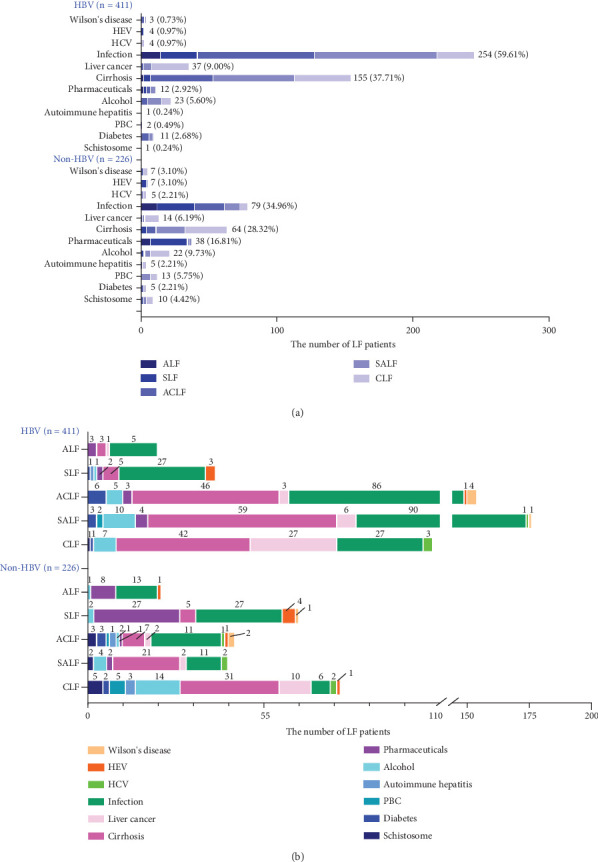
Etiological composition of patients with LF. (a) Distribution of LF etiologies in both groups. (b) Distribution of LF etiologies in each subclass. HEV, hepatitis E virus; HCV, hepatitis C virus; PBC, primary biliary cirrhosis.

According to diagnostic guidelines, infectious or immune-related diseases occurred first or LF appeared first [6]. In addition, guidelines and related studies have confirmed that ACLF (or SALF) is a complex syndrome that presents with acute decompensation, organ failure(s), and high short-term mortality and is often the focus of recent etiological studies [6, 22, 23]. LF surveillance data from the past 3 years revealed that the four leading etiological components of HBV-LF were infection (59.61%), cirrhosis (37.71%), liver cancer (9.00%), and alcohol use (5.6%), whereas the four leading causes of non-HBV-LF were infection (34.96%), cirrhosis (28.32%), medication (16.81%), and alcohol abuse (9.73%) (Figure 2(a)). In both LF groups, infection and cirrhosis in the HBV group were the main etiological components of the subclasses, particularly the ACLF, SALF, and CLF subclasses. Both groups had PBC, a history of schistosomiasis, diabetes, liver cancer, alcoholic fatty liver or cirrhosis, Wilson's disease, HEV, and hepatitis C virus (HCV). The ACLF and SALF subclasses had eight etiological components in the HBV group, and in the non-HBV group, the ACLF and CLF subclasses had 12 and 10 etiological components, respectively. However, the ALF subclass had minor etiological components in both groups (Figure 2(b)).

### 3.4. Demographic Characteristics of Patients With HBV-LF and Non-HBV-LF

After a demographic, clinical, therapeutic, and prognostic analysis of all patients with LF, the demographic indicators revealed that the overall sex ratio (male/female (M/F)) in the HBV group was 4.55, and the highest sex ratio was in the CLF subclass (8.44). The sex composition in the non-HBV group was significantly lower than that in the HBV group, and the highest sex ratio was also in the CLF subclass (1.61). Compared with patients with ALF in the corresponding group, HBV patients with SLF and CLF were significantly older; non-HBV patients with ACLF and CLF were significantly older; compared with patients with ALF in the corresponding group, the clinical indicators including the number of days hospitalized (SLF and CLF), ALT (ACLF, SALF, and CLF), and PT-INR (CLF) values in the HBV group were significantly lower; the TBil values were significantly higher in the HBV patients with SLF, ACLF, SALF, and CLF, whereas the AT III value was significantly lower in the HBV patients with SLF; the ALT (SLF and CLF) and PT-INR (SALF and CLF) values in the non-HBV group were significantly lower; the AT III value was significantly lower in the non-HBV patients with ACLF; and the number of days hospitalized (SLF, SALF, and CLF) and TBil value (SLF) in the non-HBV group were significantly higher. Compared with patients with ALF in the corresponding group, the therapeutic and prognostic indicators revealed that the incidences of hepatic encephalopathy were significantly higher in the HBV patients with ACLF (25.93%), SALF (35.19%), and CLF (31.48%). The rates of artificial liver support treatment were significantly higher in the HBV patients with ACLF (24.76%) and SALF (60.00%). The short-term (during hospitalization and within 2 weeks postdischarge) mortality rates were significantly higher in the HBV patients with ACLF (22.94%), SALF (45.29%), and CLF (26.47%). In the non-HBV group, the rate of artificial liver support treatment was significantly higher in patients with SLF (40.63%). In comparison, the short-term mortality rate was significantly higher in patients with CLF (31.58%) than in patients with ALF. Overall, consistent with the dominant subclasses, the highest mortality rates in the two groups were also in the HBV-SALF and non-HBV-CLF subclasses (Table 2).

### 3.5. Prognosis Analysis in Patients With HBV-LF and Non-HBV-LF

**Figure 3:**
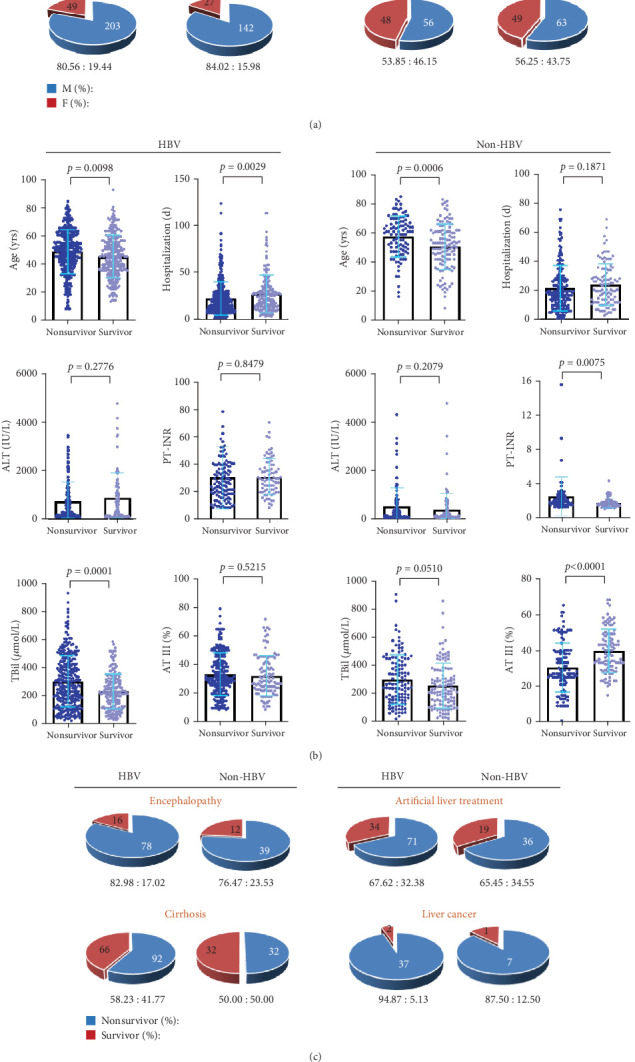
Differences in the indicators for survival and nonsurvival of patients with LF. (a) Comparison of the sex composition of surviving and deceased patients. (b) Comparisons of age, number of hospitalization days (d), alanine aminotransferase (ALT) level, prothrombin time–international normalized ratio (PT-INR), total bilirubin (TBil) level, and antithrombin III (AT III) level between survivors and nonsurvivors. (c) Comparison of the survival and mortality ratios of patients with LF with hepatic encephalopathy, artificial liver therapy, cirrhosis, and liver cancer. Statistically significant differences were analyzed via the unpaired *t*-test (Mann–Whitney test) via SPSS Statistics 22.0 software. M, male; F, female.

LF is an acute deterioration of diseases such as cirrhosis, with multiple organ failure and high short-term mortality, and some patients may even undergo liver transplantation, which requires a longer period to determine the outcome. Therefore, a telephone follow-up of survival status was carried out 6 months after discharge. The initial ALT, PT-INR, TBil, and AT III values were used as a reference for the clinical prognosis of the two LF groups. Regardless of survival status, the sex ratio (M/F) in the non-HBV-LF group was higher for men than for women, but the sex ratio was higher in the HBV-LF group (Figure 3(a)). The age of patients with HBV-LF who died was significantly higher than that of the survivors. The number of days of hospitalization among the nonsurvivors with HBV-LF was significantly lower than that among the survivors, and the initial TBil values of patients with HBV-LF who died were significantly higher than those of the survivors. In addition, the age and PT-INR values of patients who died from non-HBV-LF were significantly higher than those of the survivors, whereas AT III levels were significantly lower (Figure 3(b)). The HBV-LF group had a relatively high mortality rate of hepatic encephalopathy (82.98%) and a high rate of treatment with artificial liver support systems (67.62%). The cirrhosis mortality rate in the HBV-LF group (58.23%) was higher than that in the non-HBV-LF group (50.00%). The mortality rate of liver cancer patients in the HBV-LF group (94.87%) was higher than that of patients in the non-HBV-LF group (87.50%) (Figure 3(c)). Thus, patients who died in the HBV-LF group were older, spent fewer days in the hospital, and had higher TBil levels, which may be used to evaluate the risk of nonsurvivors. However, patients who died in the non-HBV-LF group were relatively older, had higher PT-INR values, and had lower AT III levels, which may serve as prognostic factors in the evaluation.

### 3.6. Logistic Regression Analysis of Risk Factors in Patients With HBV-LF and Non-HBV-LF

To further analyze the significance of clinical indicators on the preliminary judgment of prognosis, multiple logistic regression analysis revealed that the PT-INR of the HBV-SALF subclass (odds ratio (OR) = 7.999, 95% confidence interval (95% CI): 2.112–30.291, *p* = 0.002) was an independent risk factor affecting patient prognosis; the AT III level of the HBV-ALF (hepatitis B virus–related acute liver failure) subclass (OR = 0.724, 95% CI: 0.526–0.995, *p* = 0.047) was an independent risk factor affecting patient prognosis; and the PT-INR of the non-HBV-SLF (non-hepatitis B virus–related subacute liver failure) subclass (OR = 6.931, 95% CI: 2.315–20.750, *p* = 0.001) or non-HBV-ACLF (non-hepatitis B virus–related acute-on-chronic liver failure) subclass (OR = 37.415, 95% CI: 1.811–773.011, *p* = 0.019) was an independent risk factor affecting patient prognosis (Table S1). Therefore, PT-INR or AT III, as essential indicators, may become prognostic criteria for the HBV-SALF, HBV-ALF, non-HBV-SLF, and non-HBV-ACLF subclasses.

### 3.7. Predictive Value of ALT, PT-INR, TBil, and AT III for Prognosis in Patients With HBV-LF and Non-HBV-LF

**Figure 4:**
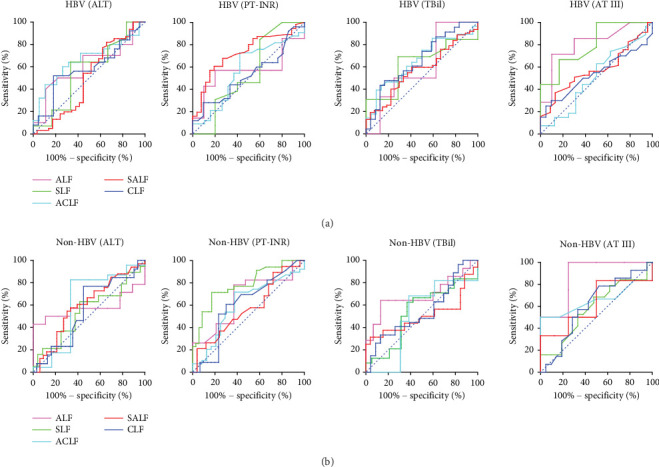
Receiver operating characteristic (ROC) curve analysis and comparison of LF outcomes based on initial ALT, PT-INR, TBil, and AT III values for each subclass. (a) ROC analyses for the HBV-LF group. (b) ROC analyses for the non-HBV-LF group. Statistically significant differences were analyzed via receiver operating characteristic (ROC) curve and area under the curve (AUC) analyses via GraphPad Prism 8.0.1 statistical software.

To analyze the value of the above prognostic factors, we needed to calculate the ROC curve of the critical indicator. The AUC of the ROC curve for the PT-INR in the HBV-SALF subclass was 0.726 (95% CI: 0.612–0.840, *p* = 0.001; sensitivity: 57.14%; specificity: 84.62%; cutoff: 2.05). Furthermore, the AUC of the ROC curve for AT III levels in the HBV-ALF subclass was 0.814 (95% CI: 0.596–1.000, *p* = 0.032; sensitivity: 71.43%; specificity: 90.00%; cutoff: 24.50%). However, the AUC values for the PT-INR in the non-HBV-SLF and non-HBV-ACLF subclasses were 0.786 (95% CI: 0.591–0.886, *p* = 0.007; sensitivity: 65.00%; specificity: 79.17%; cutoff: 1.92) and 0.609 (95% CI: 0.596–0.943, *p* = 0.004; sensitivity: 68.10%; specificity: 100.00%; cutoff: 2.11), respectively. Therefore, when the critical value of the PT-INR is 2.05 or AT III is 24.50%, this indicator has a predictive value for the prognosis of the HBV-SALF or HBV-ALF subclass, respectively. When the cutoff value of the PT-INR is 1.92 or 2.11, this indicator has a predictive value for the prognosis of the non-HBV-LF-SLF or non-HBV-ACLF subclass, respectively. Other indicators and ROC values in these subclasses did not show significant predictive values (Table S2 and Figure 4).

## 4. Discussion

To our knowledge, this study is the first systematic investigation of the etiological composition or prognostic criteria for each LF subclass. In this multicenter retrospective cohort study, the Department of Infectious Diseases of the Fifth People's Hospital of Wuxi (Wuxi Infectious Disease Hospital) is the designated LF treatment center of Wuxi City. It is the central unit of “One City and One Center for Nonbiological Artificial Liver Treatment.” The number of patients is relatively small at two participating tertiary general hospitals in southeastern China, where patients with LF are also being treated in other public hospitals. In recent decades, there have been few reports on the proportions of HBV-LF in Southeast China, so it is impossible to speculate on the morbidity or mortality trends of HBV-LF. In some studies conducted in southwestern and northern China [1, 14], the incidences of HBV-LF were 91.6% (years: 2000–2012) and 69.2% (years: 2002–2011), respectively, whereas in our study, the incidence was 64.52% (years: 2018–2020). According to statistical analysis in Southwest China, 87.3% of patients with ACLF are affected mainly by HBV infection, which is the leading cause of SALF and ACLF [14]. Current guidelines and recommendations suggest that the ACLF data include the ACLF and SALF subclasses [6, 7], and we used consistent classification criteria for calculation and comparison. In contrast, our ACLF incidence rate in this study was reduced to 80.27% (297/370, recalculated on the basis of Figure 1(c)), and our data were lower than those reported in Southwest China a decade ago [1].

The morbidity and mortality rates of LF are affected by regional economic development and medical services. Southeast China is a region with a developed economy and medical technology, so further assessment of the morbidity and mortality rates of HBV-LF in southeastern China and an understanding of the economic burden of health care are needed. Similar to previous data from northern and southwestern China [1, 14], the short-term mortality rate in patients with HBV-LF is significantly higher than that in patients with non-HBV-LF. However, our overall SS rate was higher than that before 2012 [14]. That is, timely intervention is the key to preventing deaths and obtaining successful treatment. Therefore, if the subclasses of LF can be predicted and diagnosed earlier, the timely use of artificial liver support will help reduce the incidence of hepatic encephalopathy and death [19, 24]. LF can be induced by liver diseases of various etiologies, resulting in impaired or decompensated liver function and alterations in its composition, detoxification, drainage, biotransformation function, and other abnormalities [25]. Patients with LF have different causes or trigger factors, including hepatotropic viruses, drugs, alcohol, genetic disorders, and cirrhosis. In the past 30 years, with the explosive growth of China's economy and improvements in social openness, alcohol consumption has significantly improved, and alcoholic cirrhosis has shown an increasing trend [26]. The outcome of alcohol-related cirrhosis may differ from that of HBV-related cirrhosis. Alcoholic cirrhosis is more likely to lead to hepatic encephalopathy and LF. Patients with HBV-related cirrhosis are at increased risk of liver cancer and hypersplenism [27]. Similarly, the highest proportion of the sex ratio (M/F) of LF patients in our study was 76:9 (8.44), occurring in the HBV-CLF subclass, which predominantly had underlying diseases such as cirrhosis and liver cancer. However, the incidence of non-HBV-LF is similar to that in other countries and is caused by pharmaceuticals, Chinese herbal medicines, antituberculosis drugs, infections, and alcoholism.

A prolonged PT-INR could predict thrombocytopenia and severe hepatocellular disease [28, 29]. AT III, a member of the serpin family, is synthesized primarily by hepatocytes, and its levels have been shown to be associated with liver disease in patients [30–32]. In the case of LF, not only is the clotting factor secreted by hepatocytes dysregulated, but also the anticoagulant factor secreted by hepatocytes is dysregulated, and the anticoagulant activity reflected in the AT III level is also imbalanced [32]. Our study also has several important findings. First, age, the number of hospitalization days, PT-INR, and AT III values are likely to be used as prognostic criteria for the outcomes of LF subclass patients. In addition, drug- or alcohol-induced patients with non-HBV-LF who received early nonbiological artificial liver support therapy recovered more quickly than those in the HBV-LF group. However, our previous study revealed that the success rate of nonbiological artificial liver therapy in LF individuals reached only 55.56% [33], and cirrhosis and liver cancer remain the leading causes of LF nonsurvival. Furthermore, our study revealed that SALF was the main subclass of the HBV-LF group in Wuxi, with the incidence of HBV-LF decreasing from 8.36% in 2018 to 6.24% in 2020 (Table S3).

An increasing number of studies have demonstrated the value of clinical predictive markers or mortality models for the outcomes of patients with ACLF, including the TBil, MELD score, PT-INR, and neutrophil–lymphocyte ratio (NLR) values [24, 34]. The MELD score is accepted worldwide as an effective and reliable indicator of prognosis for patients with LF and is used to assess the entire course of treatment [6, 24]. However, detailed predictive evaluations for each LF subclass are lacking. Furthermore, because the definition of ACLF varies between Eastern and Western countries, the triggering events and prognoses may also differ [4, 5]. Hence, this study is aimed at calculating meaningful diagnostic criteria by analyzing a single cause. In addition, on the basis of previous studies, the NLR has been identified as a potential marker of HBV-LF survival and prognosis [34]. Here, we calculated the ALT, PT-INR, TBil, and AT III values and found that the PT-INR value (≥ 2.05, AUC = 0.726) in the HBV-SALF subclass presented a higher ROC curve that could be used as a predictive indicator of outcomes for patients with HBV-SALF (sensitivity of 57.14%, specificity of 84.62%). In addition, the PT-INR value can also be used as a predictive indicator of outcomes for patients with non-HBV-SLF (≥ 1.92) or non-HBV-ACLF (≥ 2.11). However, the AT III level (≤ 24.50%) can be used as a predictive outcome indicator for patients with HBV-ALF. Therefore, the above initial indicator values can be used as a new reference for the prognosis of patients in each LF subclass. In summary, the AT III value as a prognostic criterion for the LF subclass is also proposed for the first time compared with previous studies.

In conclusion, this study provides evidence that the initial PT-INR or AT III value may be a potential prognostic indicator for patients in different LF subclasses. The limitation of our retrospective study is that, after these patients were divided into subclasses, the number of patients significantly decreased. In our study, the AUC of the AT III value in the HBV-ALF subclass was higher than 0.8; this indicator can be considered excellent. However, the AUC of the PT-INR value in the HBV-SALF, non-HBV-SLF, and non-HBV-ACLF subclasses was higher than 0.6, so the prognostic criteria of the PT-INR had relatively limited clinical significance in LF subclass patients. Moreover, AT III as a prognostic criterion has not been explored in the prediction of LF subclass patients. Therefore, this indicator is worth further application in the clinical tests of patients with LF. Furthermore, these indicators, combined with the LF patients' age, days of hospitalization, or TBil value, may become more objective criteria for assessing their prognosis.

## Figures and Tables

**Table 1:** Incidences and spontaneous survival (SS) rates of patients with liver failure (LF).

**Group**		**HBV**			**Non-HBV**		
**Year**		**2018**	**2019**	**2020**	**2018**	**2019**	**2020**
All patients, *n* (%)	Incidence	144 (72.36)^a^	138 (59.74)^¶^	129 (62.32)^§^	55 (27.64)^a^	93 (40.26)	78 (37.68)
	SS^b^	63 (43.75)^§^	62 (44.93)^¶^	47 (36.43)^¶^	35 (63.64)	58 (62.37)	46 (58.97)

Subclass, *n* (%)^c^	ALF	7 (4.90)	3 (2.17)	4 (3.10)	6 (10.71)	10 (10.75)	8 (10.26)
	SLF	4 (2.80)	8 (5.80)	3 (2.32)	9 (16.07)	25 (26.88)	22 (28.21)
	ACLF	27 (18.88)	50 (36.23)^¶^	51 (39.53)^¶^	5 (8.93)	10 (10.75)	6 (7.69)
	SALF	77 (53.85)	48 (34.78)^¶^	45 (34.88)^¶^	14 (25.00)	19 (20.43)	18 (23.08)
	CLF	29 (20.28)	29 (21.01)	26 (20.16)	21 (37.50)	29 (31.18)	24 (30.77)

HBV/non-HBV	ALF	1.67	0.30	0.50	N/A	N/A	N/A
	SLF	0.44	0.32	0.14	N/A	N/A	N/A
	ACLF	5.40	5.00	8.50	N/A	N/A	N/A
	SALF	5.50	2.53	2.50	N/A	N/A	N/A
	CLF	1.38	1.00	1.08	N/A	N/A	N/A

*Note:* Statistically significant differences were analyzed using the chi-square test.

Abbreviations: ACLF, acute-on-chronic liver failure; ALF, acute liver failure; CLF, chronic liver failure; HBV, hepatitis B virus; N/A, not available; SALF, subacute-on-chronic liver failure; SLF, subacute liver failure.

^a^The percentage of HBV and non-HBV cases in 2018 out of the total number of cases in that year.

^b^A statistical comparison of the corresponding composition between the HBV-LF group and the non-HBV-LF group.

^c^A statistical comparison of the subclass composition in each group by comparing with the ALF subclass.

^§^ *p* < 0.05.

^¶^ *p* < 0.01.

**Table 2:** Demographic and clinical indicators of the patients with LF.

**Group**	**Class**	**ALF**	**SLF**	**ACLF**	**SALF**	**CLF**	**Total**
(a) HBV	Male, *n* (%)	11 (78.57)	13 (86.67)	104 (81.89)	133 (78.24)	76 (89.41)	337 (82.00)
	Female, *n* (%)	3 (21.43)	2 (13.33)	23 (18.11)	37 (21.76)	9 (10.59)	74 (18.00)
	M/F ratio^a^	3.67^§^	6.50^§^	4.52⁣^∗^	3.59^¶^	8.44⁣^∗^	4.55⁣^∗^
	Age (years)^b^	48.00 ± 4.03	52.93 ± 2.52^¶^	49.84 ± 1.17	49.82 ± 0.97	53.94 ± 1.14^¶^	50.11 ± 1.03
	Hospitalization (d)^b^	23.18 ± 5.21	17.73 ± 3.63^§^	22.12 ± 1.61	26.07 ± 1.59^§^	19.62 ± 1.68^§^	24.83 ± 1.32
	ALT (IU/L)	1533.00 ± 333.5	1012.00 ± 521.10	786.60 ± 746.70^¶^	836.20 ± 697.10^¶^	198.70 ± 1335.00^∗^	766.30 ± 767.00
	PT-INR	2.50 ± 0.34	2.32 ± 0.18	2.46 ± 0.05	2.17 ± 0.33	2.06 ± 0.44^∗^	2.26 ± 0.25
	TBil (*μ*mol/L)^b^	198.46 ± 24.65	342.41 ± 24.62^&^	308.03 ± 18.05^&^	298.69 ± 17.21^¶^	207.20 ± 18.18^¶^	298.04 ± 11.42
	AT III (%)^b^	30.33 ± 2.96	39.00 ± 5.96^§^	30.96 ± 2.31	29.72 ± 1.55^§^	32.72 ± 2.90	31.70 ± 1.84
	Encephalopathy, *n* (%)	3 (5.56)	1 (1.85)	14 (25.93)^¶^	19 (35.19)^&^	17 (31.48)^&^	54 (13.14)
	Artificial liver treatment, *n* (%)	3 (2.86)	6 (5.71)	26 (24.76)^&^	63 (60.00)^&^	7 (6.67)	105 (25.55)
	Mortality^c^, *n* (%)	3 (1.76)	6 (3.53)	39 (22.94)^&^	77 (45.29)^&^	45 (26.47)^&^	170 (41.36)

(b) Non-HBV	Male, *n* (%)	13 (54.17)	29 (51.79)	9 (40.91)	29 (56.86)	45 (61.64)	125 (51.31)
	Female, *n* (%)	11 (45.83)	27 (48.21)	13 (59.09)	22 (43.14)	28 (38.36)	101 (44.69)
	M/F ratio	1.18	1.07	0.69	1.32	1.61	1.24
	Age (years)^b^	51.92 ± 3.66	54.69 ± 1.53	58.96 ± 2.86^§^	53.15 ± 2.47	57.45 ± 1.56^§^	56.18 ± 1.01
	Hospitalization (d)^b^	14.26 ± 2.33	22.91 ± 1.99^§^	14.26 ± 3.31	28.00 ± 2.88^¶^	25.68 ± 2.24^¶^	23.07 ± 1.16
	ALT (IU/L)	601.80 ± 50.29	551.50 ± 186.50^§^	646.7 ± 44.97	450.50 ± 151.20	85.19 ± 516.60^∗^	440.00 ± 161.70
	PT-INR	2.46 ± 0.56	2.27 ± 0.20	2.31 ± 0.15^&^	1.977 ± 0.49^∗^	1.94 ± 0.52^∗^	2.15 ± 0.31
	TBil (*μ*mol/L)^b^	276.43 ± 29.49	353.68 ± 22.21^§^	274.06 ± 42.79	289.01 ± 35.61	223.70 ± 20.30	281.14 ± 12.39
	AT III (%)^b^	35.86 ± 4.64	33.80 ± 2.82	26.20 ± 5.95^§^	31.33 ± 3.13	35.98 ± 2.31	34.76 ± 1.47
	Encephalopathy, *n* (%)	5 (15.15)	11 (33.33)	1 (3.03)	8 (24.24)	8 (24.24)	33 (14.60)
	Artificial liver treatment, *n* (%)	8 (12.50)	26 (40.63)^&^	7 (10.94)	15 (23.44)	8 (12.50)	64 (28.32)
	Mortality^c^, *n* (%)	14 (14.74)	22 (23.16)	11 (11.58)	18 (18.95)	30 (31.58)^¶^	95 (42.04)

*Note:* The normal reference value of antithrombin III (AT III) is 60%–120%; the normal reference value of total bilirubin (TBil) is 5–21 *μ*mol/L; the normal reference value of alanine aminotransferase (ALT) is 10–40 IU/L; and the normal reference value of the prothrombin time–international normalized ratio (PT-INR) is 5–21. The data in the table are the primary results of clinical testing at the time of admission. Statistical analyses of significance were compared to those of the ALF subclass in the corresponding group. Statistically significant differences were analyzed via unpaired *t*-tests (Mann–Whitney tests) and chi-square tests.

Abbreviations: ACLF, acute-on-chronic liver failure; ALF, acute liver failure; CLF, chronic liver failure; F, female; M, male; SALF, subacute-on-chronic liver failure; SLF, subacute liver failure.

^a^Statistical differences in sex composition compared with the non-HBV group.

^b^Mean ± SD.

^c^Nonsurvival during hospitalization or within 2 weeks after discharge.

^§^ *p* < 0.05.

^¶^ *p* < 0.01.

^&^ *p* < 0.001.

⁣^∗^*p* < 0.0001.

## Data Availability

All the data generated or analyzed during this study are included in this published article.

## Nomenclature

ACLF: acute-on-chronic liver failure
ALF: acute liver failure
AT III: antithrombin III
CLF: chronic liver failure
HBV: hepatitis B virus
HBV-LF: HBV-related liver failure
HCV: hepatitis C virus
HEV: hepatitis E virus
LF: liver failure
M/F: male/female
PBC: primary biliary cirrhosis
PT-INR: prothrombin time–international normalized ratio
ROC: receiver operating characteristic
SCLF: chronic subacute liver failure
SLF: subacute liver failure
SS: spontaneous survival
TBil: total bilirubin

## Supporting Information

Supporting Information Additional supporting information can be found online in the Supporting Information section. Table S1: Multivariate logistic regression analysis of the prognostic value of the clinical indicators for the liver failure (LF) subclasses. The risk clinical factors of patients with HBV-LF and non-HBV-LF were analyzed by multivariate logistic regression, including alanine aminotransferase (ALT), prothrombin time–international normalized ratio (PT-INR), total bilirubin (TBil), and antithrombin III (AT III). Table S2: Receiver operating characteristic (ROC) curve analysis of the prognostic value of ALT, PT-INR, TBil, and AT III values in the LF subclasses. The value of the prognostic factors was calculated by the ROC curve and area under the curve (AUC). Table S3: Estimation of the annual incidence of HBV-LF in Wuxi City. The occurrence of HBV-LF subclasses was calculated based on the number of HBV infections reported between 2018 and 2020.
