# Supplementary material for: Etiology and Prognostic Criteria for Liver Failure in Southeast China: A Multicenter Retrospective Cohort Study Between 2018 and 2020
Source: Gastroenterol Res Pract. 2024 Dec 18;2024:5512889. doi: 10.1155/grp/5512889 (PMC11669432; doi:10.1155/grp/5512889)
Supplement: Supporting Information — Additional supporting information can be found online in the Supporting Information section. Table S1: Multivariate logistic regression analysis of the prognostic value of the clinical indicators for the liver failure (LF) subclasses. The risk clinical factors of patients with HBV-LF and non-HBV-LF were analyzed by multivariate logistic regression, including alanine aminotransferase (ALT), prothrombin time–international normalized ratio (PT-INR), total bilirubin (TBil), and antithrombin III (AT III). Table S2: Receiver operating characteristic (ROC) curve analysis of the prognostic value of ALT, PT-INR, TBil, and AT III values in the LF subclasses. The value of the prognostic factors was calculated by the ROC curve and area under the curve (AUC). Table S3: Estimation of the annual incidence of HBV-LF in Wuxi City. The occurrence of HBV-LF subclasses was calculated based on the number of HBV infections reported between 2018 and 2020. [file 5512889.f1.docx]

# Supplementary material

|  |
| --- |

**Table 1 Multivariate logistic regression analysis of the prognostic value of the clinical indicators for the LF subclasses**

| Group | Indicator | Subclass | β value | S. E. | Wald | OR | 95% CI | P value |
| --- | --- | --- | --- | --- | --- | --- | --- | --- |
| 1. HBV | ALT | ALF | 0.000 | 0.001 | 0.202 | 1.000 | 0.999-1.002 | 0.653 |
|  |  | SLF | 0.000 | 0.000 | 0.346 | 1.000 | 0.999-1.001 | 0.556 |
|  |  | ACLF | -0.001 | 0.000 | 2.343 | 0.999 | 0.998-1.000 | 0.126 |
|  |  | SALF | 0.000 | 0.000 | 1.362 | 1.000 | 0.999-1.000 | 0.243 |
|  |  | CLF | 0.000 | 0.001 | 0.111 | 1.000 | 0.998-1.003 | 0.739 |
|  | PT-INR | ALF | -1.027 | 0.892 | 1.324 | 0.358 | 0.062-2.059 | 0.250 |
|  |  | SLF | -0.076 | 0.415 | 0.033 | 0.927 | 0.411-2.092 | 0.885 |
|  |  | ACLF | 0.151 | 0.203 | 0.557 | 1.163 | 0.782-1.731 | 0.455 |
|  |  | SALF | 2.079 | 0.679 | 9.366 | 7.999 | 2.112-30.291 | 0.002 |
|  |  | CLF | 0.747 | 0.603 | 1.534 | 2.111 | 0.647-6.887 | 0.216 |
|  | TBil | ALF | -0.012 | 0.013 | 0.821 | 0.988 | 0.964-1.014 | 0.365 |
|  |  | SLF | 0.004 | 0.003 | 1.440 | 1.004 | 0.998-1.010 | 0.230 |
|  |  | ACLF | 0.003 | 0.002 | 1.598 | 1.003 | 0.999-1.007 | 0.206 |
|  |  | SALF | 0.001 | 0.002 | 0.779 | 1.001 | 0.998-1.004 | 0.379 |
|  |  | CLF | 0.003 | 0.002 | 2.171 | 1.003 | 0.999-1.008 | 0.141 |
|  | AT III | ALF | -0.324 | 0.163 | 3.962 | 0.724 | 0.526-0.995 | 0.047 |
|  |  | SLF | -0.019 | 0.031 | 0.381 | 0.981 | 0.923-1.042 | 0.573 |
|  |  | ACLF | 0.005 | 0.020 | 0.071 | 1.005 | 0.966-1.046 | 0.790 |
|  |  | SALF | -0.012 | 0.020 | 0.366 | 0.988 | 0.951-1.027 | 0.545 |
|  |  | CLF | 0.014 | 0.015 | 0.932 | 1.014 | 0.985-1.044 | 0.334 |
| 1. Non-HBV | ALT | ALF | -0.002 | 0.002 | 0.356 | 0.998 | 0.994-1.002 | 0.290 |
|  |  | SLF | -0.004 | 0.007 | 0.262 | 0.966 | 0.982-1.010 | 0.609 |
|  |  | ACLF | 0.000 | 0.001 | 0.008 | 1.000 | 0.999-1.001 | 0.927 |
|  |  | SALF | 0.003 | 0.004 | 0.628 | 1.003 | 0.995-1.011 | 0.428 |
|  |  | CLF | -0.004 | 0.007 | 0.262 | .996 | 0.982-1.010 | 0.609 |
|  | PT-INR | ALF | -0.929 | 1.093 | 0.722 | 0.395 | 0.046-3.367 | 0.396 |
|  |  | SLF | 0.781 | 0.769 | 1.031 | 2.184 | 0.484-9.867 | 0.310 |
|  |  | ACLF | 3.622 | 1.545 | 5.496 | 37.415 | 1.811-773.011 | 0.019 |
|  |  | SALF | 0.422 | 0.911 | 0.215 | 1.526 | 0.256-9.095 | 0.643 |
|  |  | CLF | 0.781 | 0.769 | 1.031 | 2.184 | 0.484-9.867 | 0.310 |
|  | TBil | ALF | 0.001 | 0.005 | 0.013 | 1.001 | 0.991-1.010 | 0.909 |
|  |  | SLF | -0.003 | 0.004 | 0.678 | 0.997 | 0.990-1.004 | 0.410 |
|  |  | ACLF | 0.000 | 0.003 | 0.011 | 1.000 | 0.993-1.006 | 0.918 |
|  |  | SALF | -0.004 | 0.014 | 0.076 | .996 | 0.970-1.023 | 0.783 |
|  |  | CLF | -0.003 | 0.004 | 0.678 | 0.997 | 0.990-1.004 | 0.410 |
|  | AT III | ALF | -0.040 | 0.054 | 0.557 | 0.455 | 0.865-1.067 | 0.961 |
|  |  | SLF | 0.038 | 0.033 | 1.313 | 0.997 | 0.990-1.004 | 0.410 |
|  |  | ACLF | -0.099 | 0.061 | 2.660 | 0.906 | 0.804-1.020 | 0.103 |
|  |  | SALF | 0.051 | 0.072 | 0.495 | 1.052 | 0.914-1.211 | 0.482 |
|  |  | CLF | 0.038 | 0.033 | 1.313 | 1.039 | 0.973-1.108 | 0.252 |

Notes: LF, liver failure; HBV, hepatitis B virus; ALF, acute liver failure; SLF, subacute liver failure; ACLF, acute-on-chronic liver failure; SALF, subacute-on-chronic liver failure; CLF, chronic liver failure; ALT, alanine aminotransferase; PT-INR, prothrombin time-international normalized ratio; TBil, total bilirubin; AT III, anti-thromboplastin III.

**Table 2 ROC curve analysis of the prognostic value of ALT, PT-INR, TBil, and AT III values in the LF subclasses**

| Group | Indicator | Subclass | AUC | 95% CI | St | Cutoff value | Sensitivity (%) | Specificity (%) | *P* value |
| --- | --- | --- | --- | --- | --- | --- | --- | --- | --- |
| 1. HBV | ALT | ALF | 0.601 | 0.342-0.870 | 0.135 | 662.00 | 40.00 | 88.89 | 0.438 |
|  |  | SLF | 0.566 | 0.267-0.864 | 0.152 | 839.00 | 64.29 | 66.67 | 0.650 |
|  |  | ACLF | 0.661 | 0.499-0.823 | 0.083 | 402.00 | 60.00 | 73.68 | 0.070 |
|  |  | SALF | 0.501 | 0.360-0.643 | 0.072 | 1344.00 | 81.97 | 34.48 | 0.983 |
|  |  | CLF | 0.583 | 0.416-0.749 | 0.085 | 113.00 | 52.00 | 81.82 | 0.332 |
|  | PT-INR | ALF | 0.571 | 0.255-0.888 | 0.161 | 1.91 | 57.14 | 80.00 | 0.626 |
|  |  | SLF | 0.546 | 0.196-0.897 | 0.179 | 1.63 | 84.62 | 40.00 | 0.767 |
|  |  | ACLF | 0.572 | 0.405-0.738 | 0.085 | 1.86 | 72.73 | 57.89 | 0.393 |
|  |  | SALF | 0.726 | 0.612-0.840 | 0.058 | 2.05 | 57.14 | 84.62 | 0.001 |
|  |  | SLF | 0.528 | 0.358-0.697 | 0.086 | 2.48 | 28.00 | 90.48 | 0.749 |
|  | TBil | ALF | 0.604 | 0.293-0.915 | 0.159 | 227.50 | 100.00 | 37.50 | 0.519 |
|  |  | SLF | 0.632 | 0.380-0.884 | 0.129 | 304.00 | 69.23 | 71.43 | 0.342 |
|  |  | ACLF | 0.657 | 0.504-0.809 | 0.078 | 402.40 | 39.29 | 90.91 | 0.059 |
|  |  | SALF | 0.576 | 0.452-0.699 | 0.063 | 260.00 | 59.68 | 59.26 | 0.259 |
|  |  | CLF | 0.665 | 0.501-0.820 | 0.079 | 244.00 | 47.83 | 83.33 | 0.528 |
|  | AT III | ALF | 0.814 | 0.596-1.000 | 0.112 | 24.50 | 71.43 | 90.00 | 0.032 |
|  |  | SLF | 0.806 | 0.577-1.000 | 0.117 | 29.00 | 66.67 | 83.33 | 0.052 |
|  |  | ACLF | 0.514 | 0.330-0.698 | 0.094 | 23.50 | 62.96 | 50.00 | 0.880 |
|  |  | SALF | 0.577 | 0.449-0.705 | 0.065 | 19.50 | 36.84 | 86.96 | 0.283 |
|  |  | CLF | 0.539 | 0.356-0.723 | 0.094 | 45.50 | 30.00 | 90.48 | 0.667 |
| 1. Non-HBV | ALT | ALF | 0.579 | 0.339-0.820 | 0.123 | 754.50 | 42.86 | 100.00 | 0.529 |
|  |  | SLF | 0.544 | 0.365-0.723 | 0.091 | 460.50 | 63.16 | 58.33 | 0.625 |
|  |  | ACLF | 0.616 | 0.308-0.924 | 0.157 | 47.50 | 82.61 | 66.67 | 0.389 |
|  |  | SALF | 0.597 | 0.458-0.736 | 0.071 | 194.50 | 57.58 | 66.67 | 0.176 |
|  |  | CLF | 0.558 | 0.375-0.742 | 0.094 | 48.00 | 76.92 | 54.84 | 0.550 |
|  | PT-INR | ALF | 0.671 | 0.435-0.908 | 0.121 | 1.70 | 83.33 | 55.56 | 0.189 |
|  |  | SLF | 0.739 | 0.591-0.886 | 0.075 | 1.92 | 65.00 | 79.17 | 0.007 |
|  |  | ACLF | 0.789 | 0.575-1.000 | 0.109 | 1.99 | 69.23 | 100.00 | 0.089 |
|  |  | SALF | 0.567 | 0.384-0.749 | 0.093 | 2.15 | 88.24 | 30.43 | 0.477 |
|  |  | CLF | 0.593 | 0.386-0.799 | 0.105 | 1.85 | 66.67 | 62.96 | 0.411 |
|  | TBil | ALF | 0.691 | 0.485-0.896 | 0.105 | 395.60 | 64.29 | 86.67 | 0.081 |
|  |  | SLF | 0.537 | 0.358-0.716 | 0.091 | 252.50 | 62.50 | 63.16 | 0.678 |
|  |  | ACLF | 0.507 | 0.286-0.728 | 0.113 | 357.00 | 68.18 | 61.54 | 0.946 |
|  |  | SALF | 0.506 | 0.300-0.712 | 0.105 | 382.70 | 31.25 | 96.15 | 0.948 |
|  |  | CLF | 0.546 | 0.383-0.709 | 0.083 | 272.00 | 33.33 | 86.96 | 0.579 |
|  | AT III | ALF | 0.875 | 0.608-1.000 | 0.136 | 42 | 100.00 | 75.00 | 0.083 |
|  |  | SLF | 0.583 | 0.402-0.763 | 0.092 | 32.50 | 68.42 | 52.38 | 0.371 |
|  |  | ACLF | 0.653 | 0.311-0.995 | 0.175 | 25.00 | 50.00 | 100.00 | 0.379 |
|  |  | SALF | 0.625 | 0.302-0.949 | 0.165 | 26.00 | 83.33 | 50.00 | 0.439 |
|  |  | CLF | 0.607 | 0.415-0.799 | 0.098 | 26.50 | 78.57 | 47.62 | 0.289 |

Notes: HBV, hepatitis B virus; ROC, receiver operating characteristic; AUC, area under the curve; 95% CI, 95% confidence interval; St, Standard error; ALF, acute liver failure; SLF, subacute liver failure; ACLF, acute-on-chronic liver failure; SALF, subacute-on-chronic liver failure; CLF, chronic liver failure; ALT, alanine aminotransferase; PT-INR, prothrombin time-international normalized ratio; TBil, total bilirubin; AT III, anti-thromboplastin III.

**Table 3 Estimation of the annual incidence of HBV-LF in Wuxi city**

| Class | 2018 | 2019 | 2020 | Average |
| --- | --- | --- | --- | --- |
| HBV infected individuals | 1041 | 1059 | 929 | 1009.67 |
| ALF, n (%) | 3 (0.29) | 2 (0.19) | 0 | 1.67 (0.17) |
| SLF, n (%) | 4 (0.38) | 5 (0.47) | 3 (0.32) | 4 (0.40) |
| CALF, n (%) | 16 (1.54) | 18 (1.70) | 15 (1.61) | 16.33 (1.62) |
| SALF, n (%) | 37 (3.55) | 22 (2.08) | 24 (2.58) | 27.67 (2.74) |
| CLF, n (%) | 27 (2.59) | 36 (3.40) | 16 (1.72) | 26.33 (2.61) |
| Total incidence, n (%) | 87 (8.36) | 83 (7.84) | 58 (6.24) | 76 (7.53) |
|  |  |  |  |  |

Notes: Combined with the total number of hepatitis B virus (HBV) infections in Wuxi collected by the Wuxi Municipal Statistical Information Center, the trend of HBV-liver failure (LF) incidence in each year was analyzed. ALF, acute liver failure; SLF, subacute liver failure; ACLF, acute-on-chronic liver failure; SALF, subacute-on-chronic liver failure; CLF, chronic liver failure.
